# The Historic Materials and Structures Due to the Aspect of Their Actual Challenges

**DOI:** 10.3390/ma16062302

**Published:** 2023-03-13

**Authors:** Dariusz Bajno, Agnieszka Grzybowska, Ireneusz Trzyński

**Affiliations:** 1 Department of Building Structures, Faculty of Civil and Environmental Engineering and Architecture, Bydgoszcz University of Science and Technology, 85-796 Bydgoszcz, Poland; 2 Ag-Cel Laboratory, ul. Bydgoska 14, 89-620 Chojnice, Poland

**Keywords:** staircases exploitations, buildings diagnostics, historic buildings, concrete/ceramic staircases, in-situ/laboratory tests, physical/mechanical parameters

## Abstract

The subject of the article is to assess the suitability of over materials over a hundred years old that are embedded in historic building structures in conditions of contemporary utility challenges in residential and public buildings. It is based on an example of technical condition evaluation of a ceramic staircase erected in 1840 and a structure of two reinforced concrete staircases from the year 1910. As a part of in-situ and laboratory tests, the physical and mechanical parameters of unique historical materials (brick, concrete) were determined. Then the conditions for their incorporation were inventoried and determined in order to save the unique material and technical solutions used in the first half of the 19th century and the first decade of the 20th century. The article is a summary of the research and analyzes carried out in terms of proper handling of historical materials, buildings and their elements that could still fulfill their original function and be a witness to the history of a certain era. Both a research case and an application case are described here. It will allow for the continuation of these studies directly in the facility, thus assessing the effectiveness and suitability of such methods for use in similar or other situations. The aim of this approach was to introduce a non-invasive reinforcing technique that would not change the valuable and authentic appearance of these historic structures. It would also not change their static schemes, and at the same time would ensure their proper load-bearing capacity, bearing in mind that the materials used here are not equivalent to current regulations and standards.

## 1. Introduction

The inspiration to take up the presented topic was the inquiry of the Provincial Office for the Protection of Monuments about the possibility of leaving and further to use the historic staircases in renovated buildings in new conditions. Designers of renovation, strengthening and modernization works had assigned these structures to demolition due to the low strength of concrete and because of currently not used technologies [1,2,3,4,5,6,7,8,9,10,11,12,13,14,15,16,17,18,19,20,21,22,23,24,25].

Authors of the article decided to evaluate these materials and use them in structures in terms of suitability for today’s construction and operational requirements. As it was mentioned above, the subject of the research was over a hundred-year-old materials, of which the structure of three staircases marked as K1, K2 and K5 (ceramic) were made. The main idea was to leave them for further use in a new function of the building, i.e., the Centre for Vocational Education, and indicating the scope and solutions of their possible reinforcements. The research, the accurate inventory of three subjective structures and also the research of the elaboration [26,27,28,29,30,31,32] were used to carry out the static and strength analysis and to indicate the reinforcement zones where the materials did not show sufficient load-bearing capacity.

The ceramic (K5) and concrete (K1, K2) staircases responsible for vertical communication in buildings have been so far used as fully-fledged load-bearing elements in a public utility facility (hospital facility with clinics), until it was shutdown. An object of this rank had to be regularly inspected, but at the time of performing these elaborations [1,2], their authors did not have such documents at their disposal. Inspection of the above-mentioned structures, carried out in December 2021 and April 2022, did not find any deformations and damage to these structures in terms of excessive deflection, cracks or even scratches exceeding the limits considered acceptable, i.e., 0.3 mm. Initially, the condition of the structure was considered to be in a good technical condition and suitable for further exploitation.

In terms of diagnostics, it is also important to refer to the applicable regulations and standards. For calculating the load-bearing capacity of the staircase structure, Polish Standards were used [20,21,22,23,24,25], older than the current ones, which were considered to be closer to the historical solutions at the time when the building was erected with no obligatory standards. An example is the class of concrete. In the current standards, the lowest strength class is C12/15 (former designation B15), while at the time of the staircase construction, these were classes from the B3 to B5 range, in special cases B7 (B7.5). Such classes can still be found with the Polish Standard from year 1976 [22].

## 2. Materials and Methods

The aim of the work was to make the research of building materials (shown in Table 1, Table 2 and Table 3) and to determine the static schemes that were necessary to perform the calculations that allows to verify the load capacity of the staircases, e.g., in order to propose the appropriate reinforcements, where the bearing capacity has been exhausted. As a result of the analysis, a secondary aim emerged, i.e., to propose reinforcements for areas that were exhausted in terms of their bearing capacity. As another method, calculations performed on the basis of the strength results of tested materials should be indicated. Calculations were made with our own algorithms and calculation programs based on FEM, such as Autodesk Robot Structural Analysis. Conducted moisture tests of bricks and concrete of the staircases’ structures showed an air-dry condition of moisture, in which the mass moisture content of the mentioned materials did not exceed 3%. No traces of moisture were found to have been caused by leaky roofs or capillary humidity rise in those staircases.

### 2.1. Loads

Both values of permanent and functional loads were adopted on the basis of Polish Standards [20,21], with corresponding load factors. This corresponded to the values of the loads assumed for the design of the structure during the erection period of the buildings. The usable load of staircases was assumed in accordance with “Table 2a Communication spaces” [21], with a value of 4.0 kN/m^2^. It is the number of loads required to be transferred on communication areas in office buildings, schools, research institutes, banks and medical clinics. The specifics of the above-mentioned rooms do not differ much from those in hospitals and prison buildings, where the required operational loads are 1.0 kN/m^2^ less, the value being 3.0 kN/m^2^. This load is classified as a long-term, in fact it will be a short-term or even temporary load. In the combination of loads, only a part of it (its short-term value according to PN-82/B-02003) described by the coefficient ψd = 0.35 could be assumed, but its full value was taken into account in the verification calculations due to the fact that these structures have already passed the technical service life that were assumed for them (over 100 years). The structures of all three staircases have been verified due to capacity of the load-bearing characteristic value of 4.0 kN/m^2^.

### 2.2. The Perfomance Technologies of Staircases

#### 2.2.1. Concrete and Iron (Steel) for Reinforcement Bars—K1 and K2 Reinforced Concrete Staircase

**Figure 1:**
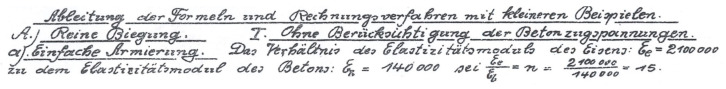
A fragment of a German-language manuscript that describes properties of materials [33]. Translation below.

The first tests of concrete compressive strength [30,31,32], carried out on three samples, revealed a very low class of concrete, of the order of 9.2 MPa ÷ 12.3 MPa (the middle value was 10.0 MPa), which did not allow for classification within the concrete class currently in construction. Nevertheless, in first three decades of the 20th century, concrete classes with kb = 30, 35, 45, 45, 50 kg/cm^2^ (currently they should be defined as B3 ÷ B5, there is no reference to Eurocode 6) were commonly used in concrete structures and concrete reinforced with iron (steel) bars [25,33]. In the first decade of the 20th century, design studies of concrete structures at that time, including stairs, class B7.5 (E_b_ = 140,000 kg/cm^2^) concrete was also used and iron for reinforcement bars with E_e_ = 210,000 kg/cm^2^ [33] fragment of the description below—Figure 1 (original, translated from German language).

*“A derivation of formulas and calculation methods with small examples*

*A. Pure bending I. without considering concrete tensile stresses.*

*(a) Simple reinforcement. The ratio of the modulus of elasticity of iron ε_e_ = 2,100,000 kg/cm^2^ to the modulus of elasticity of concrete ε_b_ = 140,000 kg/cm^2^*

*ε_e_/ε_b_ = n = 2,100,000/140,000 = 15”*

The research of concrete structures from the beginning of 20th century indicated much higher strength parameters than assumed by their original designers. An example of this is the research carried out by another laboratory, which has checked the mechanical parameters of concrete and bricks by the request of the following article authors.

Basing on the compression research of 3 concrete samples according to [30], it was possible to assume the B12.5 concrete class, which was used quite often in Poland and was even used in the second half of the 20th century to design and manufacture concrete and reinforced concrete structural elements. Concrete testing for the K1 staircase (presented in the following sections), using both the non-destructive and laboratory methods, indicated much higher compressive strength classes of concrete [28]. On their basis, it was assumed that on average it will be class C20/25 (B25), but for the verification calculations, lower parameters of structural concrete, corresponding to class C12/15 (B15), were adopted. Additionally, the load-bearing capacity of these structures was checked, assuming that they were made of B12.5 class concrete. Additionally, a series of “in situ” research has been carried out using the “Schmidt’s hammer” sclerometer, not simply to estimate the concrete class, but to evaluate the homogeneity in its structure.

It is similar with reinforcing steel (iron, in this case, probably cast). According to the study [25], the immediate tensile strength of steel with a modulus of elasticity E = 2,100,000 kg/cm^2^ ranges from 3700 kg/cm^2^ (370 MPa) to 5800 kg/cm^2^ (580 MPa). According to the studies [34], the tensile strength of cast steel was in the range of Rm = 370 ÷ 450 MPa, so it was practically consistent with the source cited earlier. These steels can be considered as the equivalent of St3S steel according to PN-88/H-84020 with Rm = 360 ÷ 490 MPa. This is also confirmed by the research [31], where basing on the measurement of steel hardness, the tensile strength Rm = 235 MPa was assigned to it.

**Figure 2:**
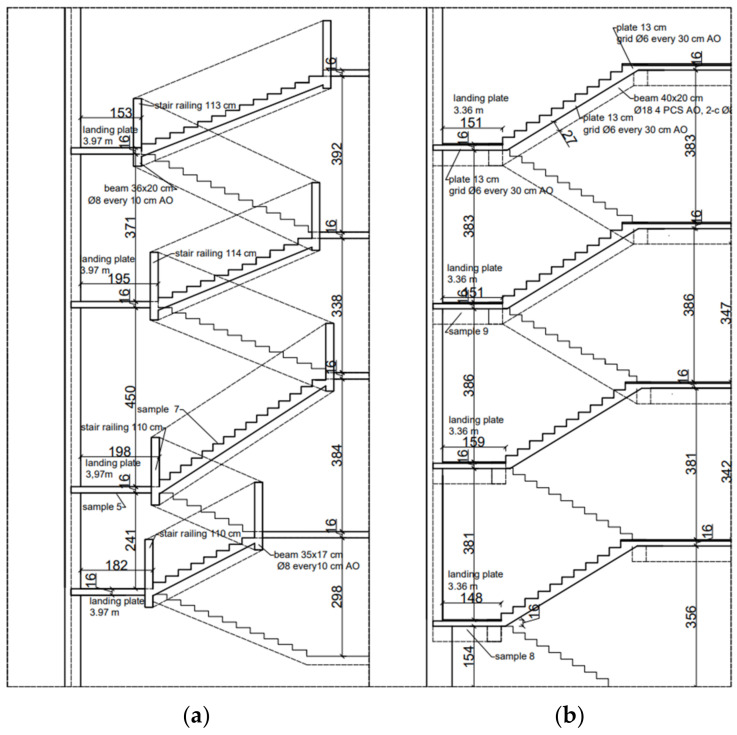
A view of staircases (**a**) staircase K1, (**b**) staircase K2.

**Figure 3:**
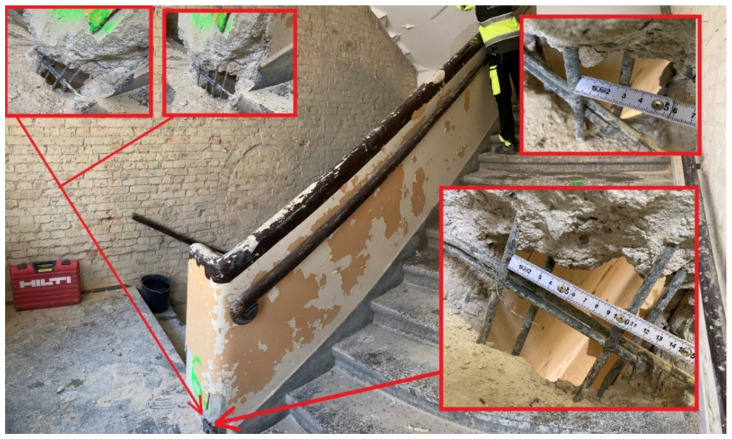
A view of a staircase with a sample 8 location.

This section presents the results of research carried out for the structural elements made of reinforced concrete of the K1 staircase, which also had a reinforced concrete railing that served as the stringers of the running plates. The structural thickness of the running plate and landing is 13 cm, and the utility layer is 3 cm thick terrazzo (Figure 2a). The reinforced concrete balustrade is monolithised (composite) with the stair tread and constitutes the above-mentioned cheek beam. Figure 2a and Figure 3 shows the place of collecting the tested samples. Representative sample No. 7 was collected in the form of a core in the staircase, which was then tested in a strength press. Sample No. 5 was taken from the landing. The test was carried out in the Pilot 4 testing machine in accordance with the applicable standards, the results are presented in Table 2.

**Figure 4:**
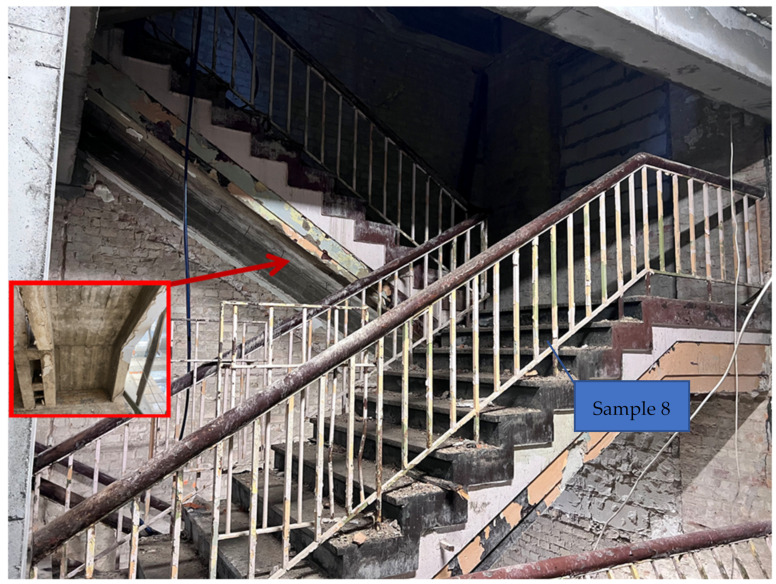
A view of the K2 staircase.

Another tested element of vertical communication was the K-2 staircase, which is fully monolithic. It was built by the P-3 slab (13 cm thick, with 16 cm thick landing and the following layers: 13 cm reinforced concrete slab and 3 cm of finishing layers). On both sides, it rests on 2 B-3 reinforced concrete stringers. In the level of the platforms the slabs rest on the new supporting structure, and the other side on the wall. They were connected with a massive B-3.1 reinforced concrete beam in the space between the running plates of the landing, probably in order to stiffen the entire structure. The staircase diagram is shown on Figure 2b and Figure 4. Sclerometric and destructive tests were carried out on the concrete of running and landing slabs as well as reinforcement iron (steel) inserts.

It was not necessary to carry out a statistical analysis in this example and plan the experiment, because the samples were made to verify those presented in previous studies. The general aim was not a new experiment, but to determine the parameters of the existing structure, which was associated to limit the number of taken samples.

**Figure 5:**
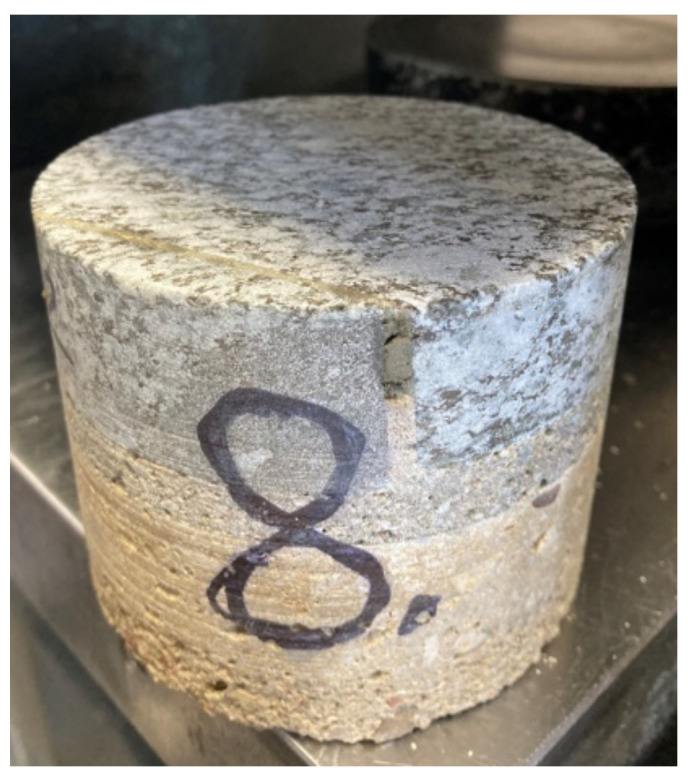
Core concrete sample No. 8 (borehole diameter 98 mm).

On Figure 2b. The diagram of K2 staircase indicates the place of collecting the research samples. Sample No. 8 was taken from the landing slab. A core sample was taken (Figure 5) and then tested in a strength press. Representative sample No. 9 was taken from the landing.

The tests of taken samples were carried out in the Pilot 4 testing machine in accordance with the applicable standards. The obtained results are presented in Tables in Section 3.

#### 2.2.2. Ceramic K5 Staircase

In the first decades of the 20th century, ceramic bricks were commonly used, the so-called clinker—with compressive strength Kc = up to 640 kg/cm^2^, the so-called class I masonry with Kc = up to 160 kg/cm^2^ (16 MPa), class II with Kc = ~100 kg/cm^2^ (10 MPa) and sand-lime with Kc = ~180 kg/cm^2^ (18 MPa). In fact, the strength class of the bricks was much higher, which is confirmed by most of the research carried out today. The current condition of masonry structures depends on the conditions in which they were used, as well as whether the maintenance and repair procedures have been carried out or not. The collected samples of bricks (walls) from the walls of the building [32] have shown their examination a considerable range of strength, even up to 30 MPa (in a dry condition, i.e., with a mass humidity of ≤3%). The average value of this strength was set at 18.1 MPa. A similar result was obtained during laboratory tests [28], f = 28 MPa. The ceramic structures of the staircases did not show any moisture content, so the level of moisture in the bricks corresponded to the dry condition (acceptable humidity).

**Figure 6:**
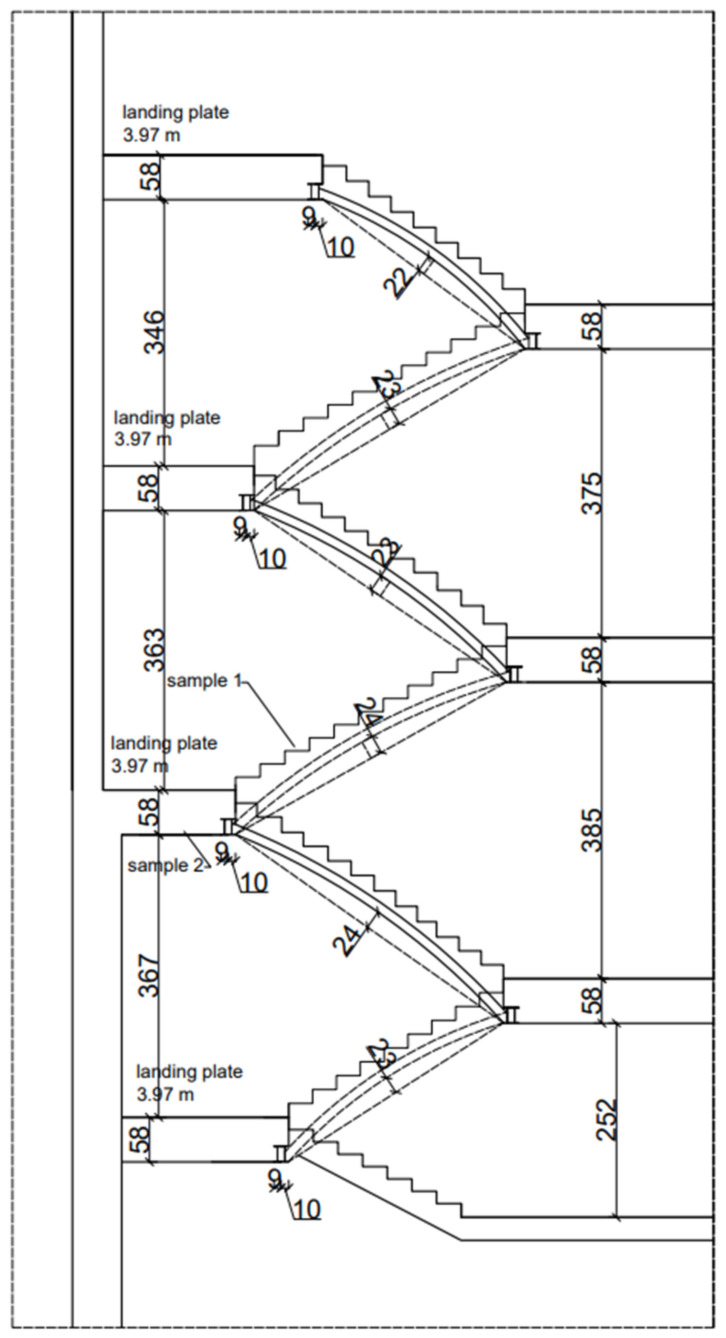
A view of K5 staircase.

**Figure 7:**
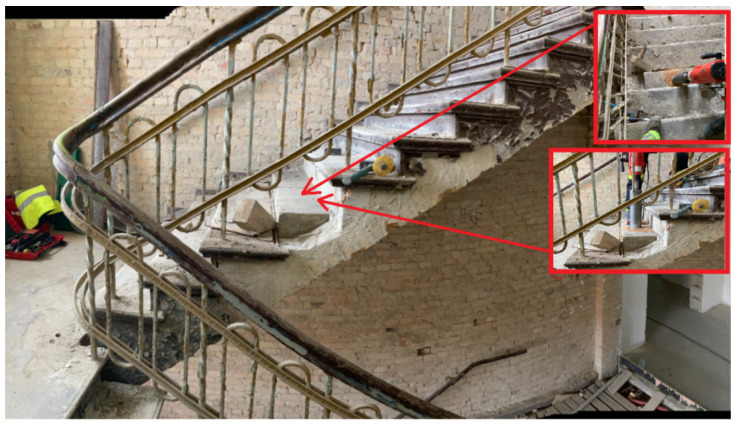
A view of a staircase with sample 1 location.

This part of article describes tests of the ceramic and vaulted structural elements of the K5 staircase, which also served as a starting point for the verification calculations. The load-bearing structure of the staircase consists of ceramic running (bricks and mortar), landing and landing plates based on walls and iron (steel) beams. The main subject of the research and analysis of the structure in question if a brick was a running plate (vault with a thickness of ½ brick—12 cm) with an arc arrow up to 24 cm, shown above (Figure 6 and Figure 7), on which the places of sampling for strength research were marked. Representative sample no. 1 (core borehole) was taken in the course of the staircase.

**Figure 8:**
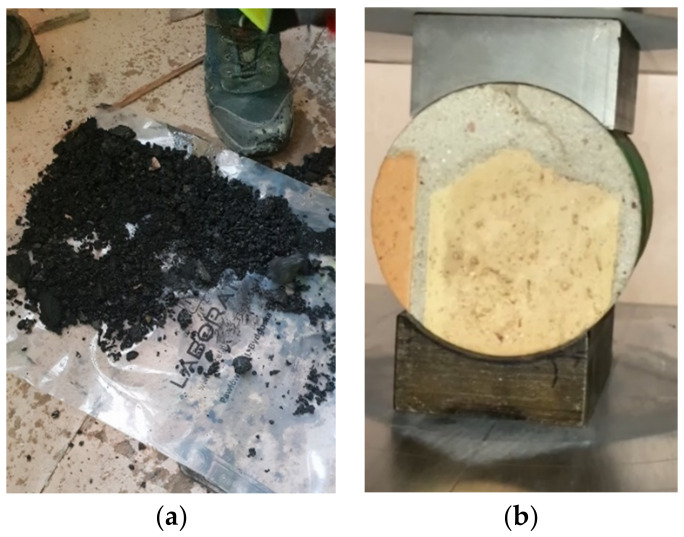
Bitumen sample (**a**) and a core cross-section (borehole diameter 98 mm) (**b**).

Representative sample No. 2 was taken from the landing (Figure 8). The sample tests were carried out in the Pilot 4 testing machine in accordance with the applicable standards. The obtained results are presented in Table 1.

**Table 1:** The strength test results for samples no. 1 and 2.

Sample No.	Sample Weight [kg]	Average Height of the Prepared Sample [mm]	Average Length of the Prepared Sample [mm]	Compression Strength f [N/mm^2^]
1 (average-horizontal test)	1.387	98	98	28.05
1a	1.385	97	98	27.09
2a	1.385	98	97	28.15
2b	1.387	97	99	28.20
1d	1.387	98	98	28.23
1e	1.387	98	98	28.31
1f	1.387	98	98	28.05
2 (average-vertical test)	0.867	122	98	5.00
2a	0.872	123	99	5.05
2b	0.863	121	98	4.93
2c	0.866	122	97	4.83
2d	0.867	122	97	5.09
2e	0.869	123	99	5.02
2f	0.863	121	97	5.09

Slag/the so-called slag mixed with tar, for which the bulk and tapped bulk densities were determined to be 0.89 g/cm^3^ and 1.08 g/cm^3^, respectively.

## 3. Results

For each staircase, samples 8a–f were taken in the running plate and samples 9a–f in the landing plate. Results are presented in this section.

### 3.1. K5 Ceramic Staircase

The obtained results are presented in Table 1—there are two representative samples (samples 1 were taken within the running plate, samples 2 were taken within the landing plate), and each had results a–f. Tests were done on different sub-samples from the same brick, which were taken in multiple places.

**Figure 9:**
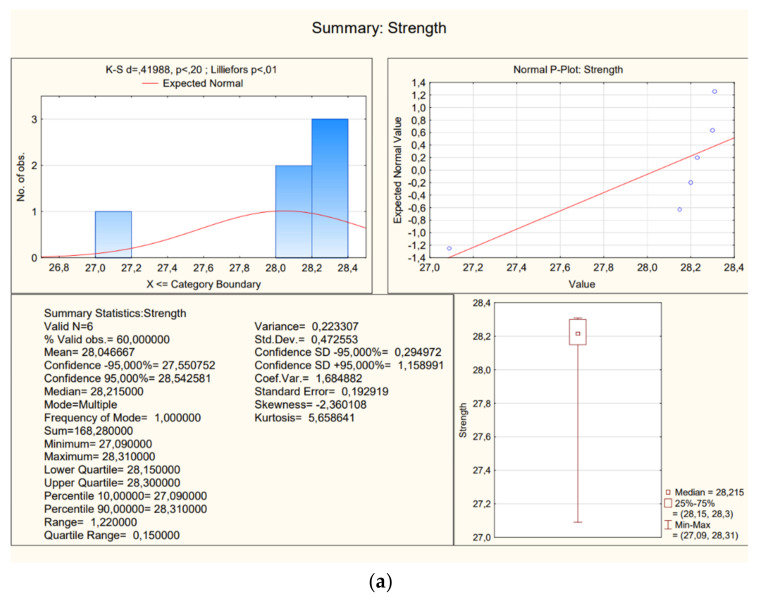

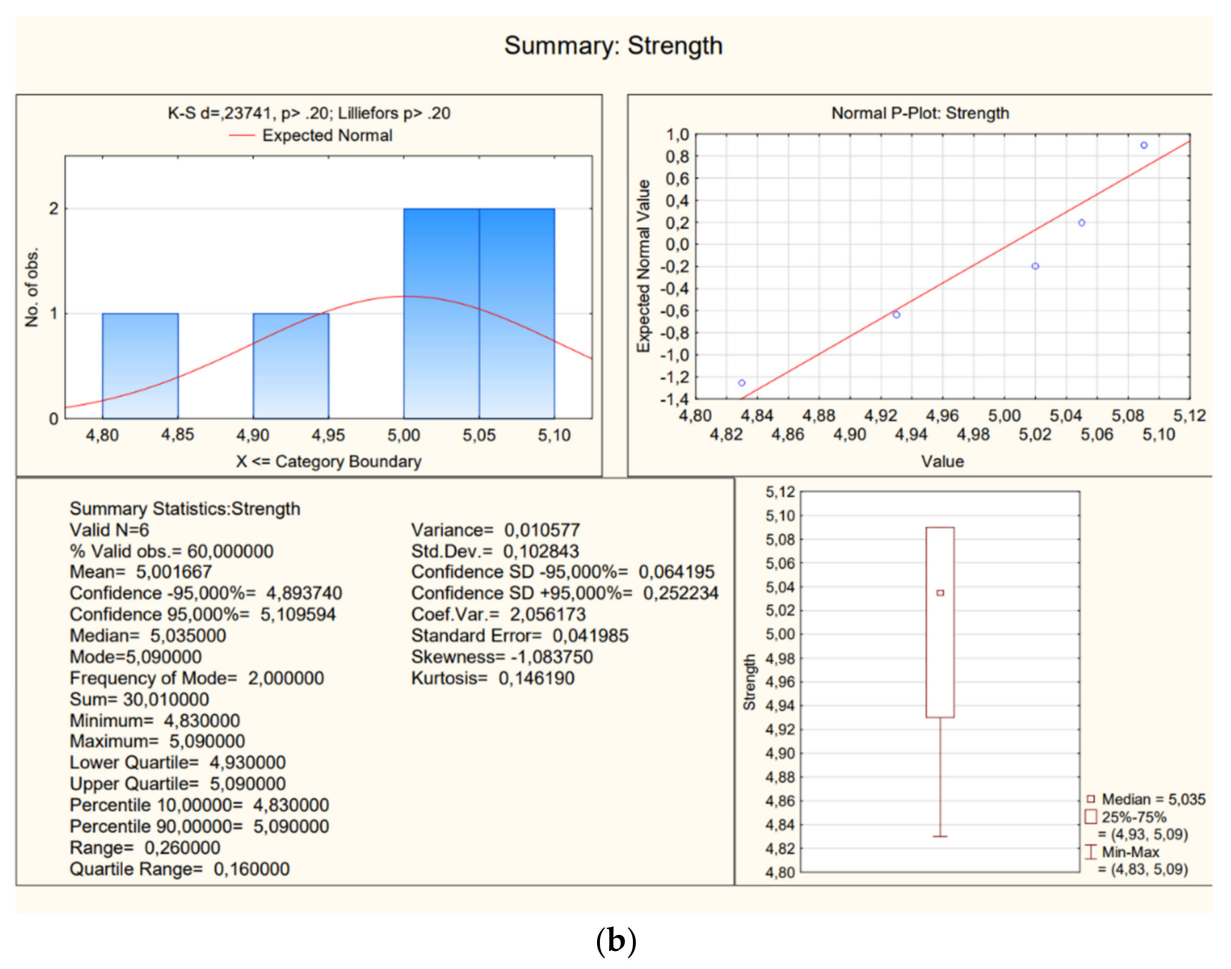
A view of statistics (**a**) samples 1a–1f; (**b**) samples 2a–2f.

Basic statistics presented in the following figures were carried out for the research results (Figure 9).

**Figure 10:**
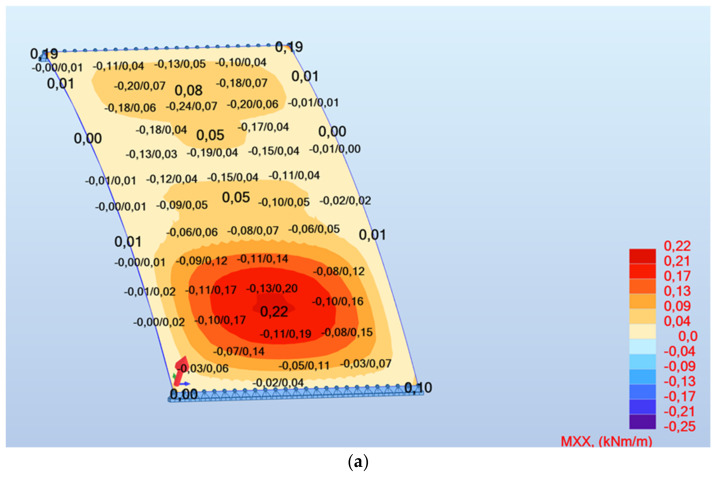

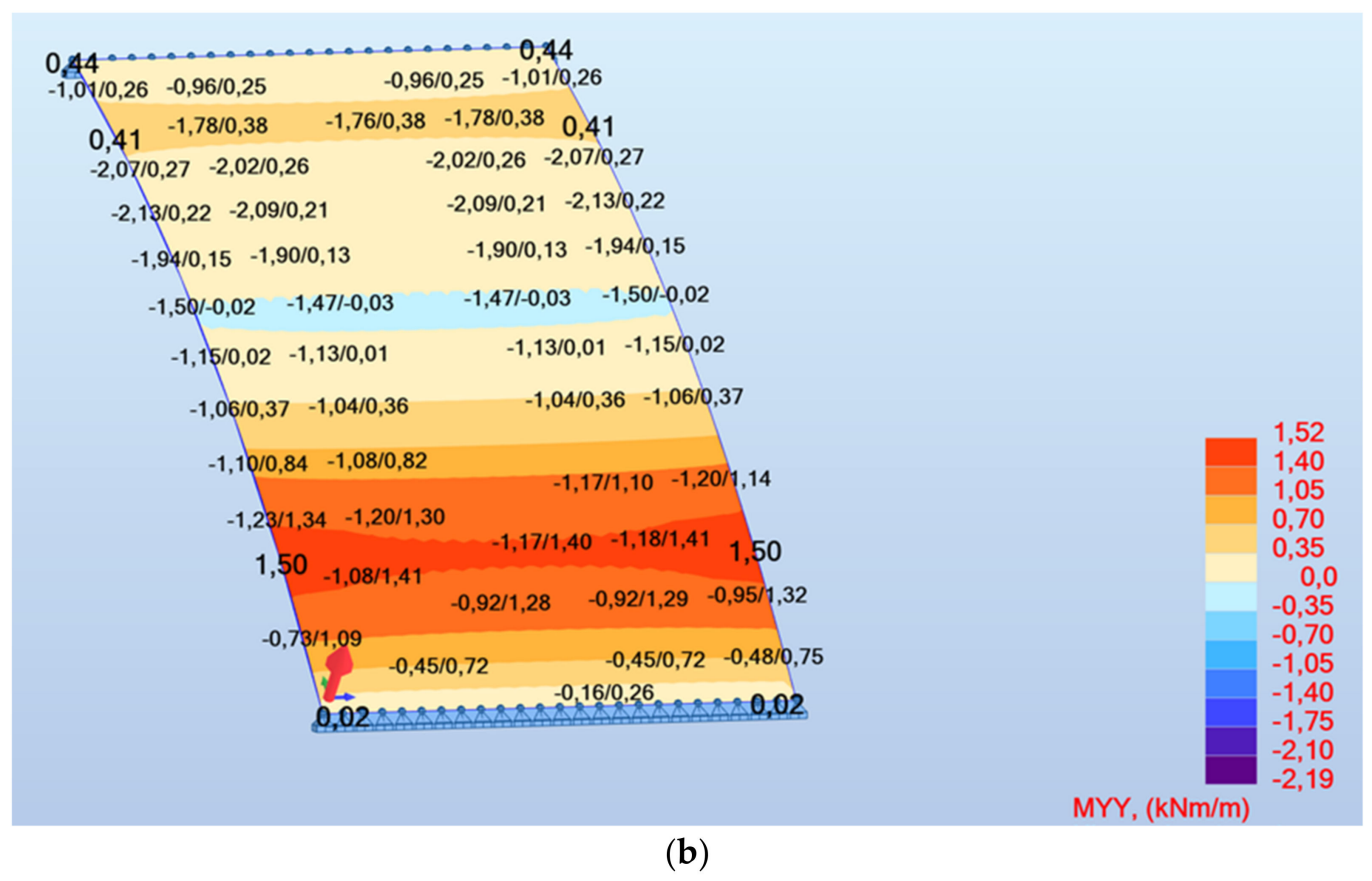
A view of the dialog box (**a**) map of XX moment, (**b**) map of YY moment.

The values of internal forces in the vault were determined using Robot Autodesk Structural Analysis. The selected calculation results are shown on Figure 10, which shows an example of the calculation results.

As mentioned in the studies [26,27], the ceramic running plates are vaulted structures and as vaults they did not require separate reinforcement. To calculate the stresses and check the bearing capacity of the above-mentioned, the structure was verified with the aforementioned Robot Autodesk Structural Analysis program. Whereas the calculation results were verified with the Rama R2D2 Intersoft program and the proprietary algorithm [26], created in the Mathcad 7 program, based on standards [20,21,24]. In the study [28], the compressive strength of bricks was determined at the level of 28 MPa, while in the study [32] at an average of 18.1 MPa. Finally, the compressive strength of bricks was assumed for the verification calculations, equal to 15 MPa and mortar of the class (brand) of 5 MPa (cement-lime mortar, as it was present here). The principle of the correct shaping of the vaults was to adjust their shape (the arch height f) in such a way that the pressure line was within the contour of the loaded element. In this case, we deal locally with tensile stresses in the ceramic cross-section of the running plate, causing the pressure line to exceed the cross-section.

### 3.2. K1 Reinforced Concrete Staircase

The obtained results are presented in Table 2—there are two representative samples (samples 1 were taken from the running plate, samples 2 were taken from the landing plate), and each had results a–f. Tests were done on different sub-samples from the same concrete, which were taken in multiple places.

**Table 2:** Strength test results for samples no. 5a–f and 7a–f.

Sample No.	Sample Weight [kg]	Average Height of the Prepared Sample [mm]	Average Length of the Prepared Sample [mm]	Strength on Compression f [N/mm^2^]
5 (average)	1.480	98	85	36.4
5a	1.45	99	83	36.3
5b	1.5	96	86	35.9
5c	1.49	97	86	37
5d	1.48	98	84	36.5
5e	1.48	98	86	36.4
5f	1.46	99	84	36.2
7 (average)	0.867	98	98	22.3
7a	0.859	95	96	22.3
7b	0.873	100	94	22
7c	0.862	99	97	22.3
7d	0.865	97	99	23
7e	0.866	98	99	21.9
7f	0.875	98	100	22.5

**Figure 11:**
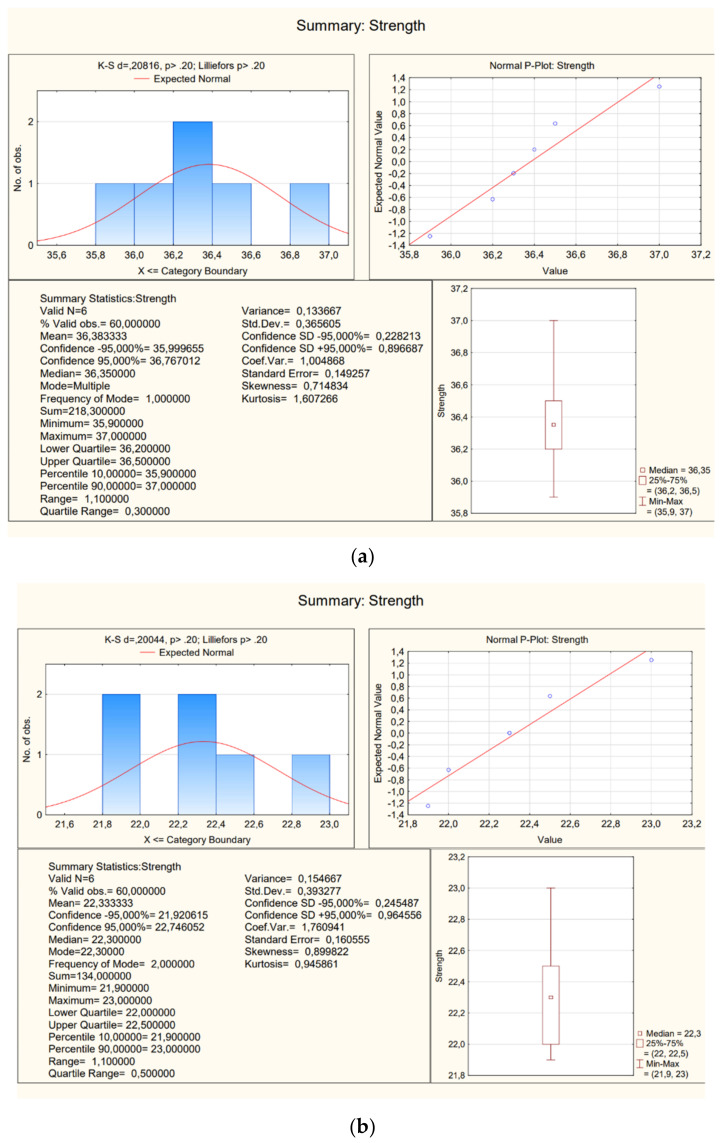
A view of statistics (**a**) samples 5a–f; (**b**) samples 7a–f.

Basic statistics presented in the following figures (Figure 11) were carried out for the research results.

Strength tests of 3 samples (tested in [30]) of concrete used for the structure of stairs in compression according to [30] has shown its very low class in relation to the current requirements B10 ÷ B12.5; therefore, it was initially considered that these structures had insufficient bearing capacity of running plates and landings and should be dismantled according to the owner and project manager. The subsequent research carried out on a much larger number of samples, taken from various places of the staircase, showed higher strength parameters of concrete; therefore, as already mentioned above, the verification calculations were carried out for two concrete classes B15 (C12/15) and B12.5 (without modern concrete). The calculation results obtained for the concrete class B12.5 were assumed as “safer”, despite the fact that the tests indicated a much higher class. In [26], the actual number of reinforcement bars was compared to the computationally required amount. This required an additional, more detailed diagnosis on the site before making a decision to leave the existing condition for further use or to supplement the reinforcement.

The structure of the stairs is made of a running plate (13 cm thick) supported on the longitudinal wall of the staircase and a running string, which is also a reinforced balustrade. In the assumptions for the calculations, it was assumed that the balustrade is entirely a load-bearing stair cheek beam. It was also assumed that the P-1 running plate is freely supported on both sides, although in fact it can be fixed on one side in the above-mentioned “cheek”. Sclerometric and destructive tests were carried out on the concrete of running and landing slabs, as well as reinforcement iron (steel) inserts. Such an assumption would significantly reduce the span moment, the amount of deflection and the computationally required amount of reinforcement bars. The performed verification calculations showed that the inventoried number of reinforcing bars should be sufficient to transfer the moment, but their spacing does not meet the current code requirements [22], because it is too large. The permissible maximum bar spacing according to PN [22] should be the smaller of the two values: 25 cm or 1.2 h = 15.6 cm, i.e., much smaller than the actual one, set at 24 cm. In this situation, it was proposed to strengthen the boards from the bottom with one layer of PBO mesh applied on the mineral matrix. General requirements for the reinforcement of ceramic and concrete structures with the use of composite materials are given in point 3. The P-2 landing slab is supported on the wall and on the landing beam. The spacing (17 cm) and diameters of the bearing bars (ø8—the equivalent of S235 class steel) are computationally and structurally acceptable here, and only these values should be confirmed directly on the construction site before starting the works. The permissible maximum bar spacing according to PN [22], as stated above, should be the smaller than the two values 25 cm or 1.2 h = 15.6 cm (the actual spacing of ~17 cm is close to the permissible values). If such bar diameters and spacing are confirmed, it will be possible to leave the structure of the plates without additional reinforcements. The B-1 cheek beam, which is also a stair railing (balustrade), has a sufficient load-bearing capacity assuming the work of the entire cross-section, i.e., about 100 cm high.

**Figure 12:**
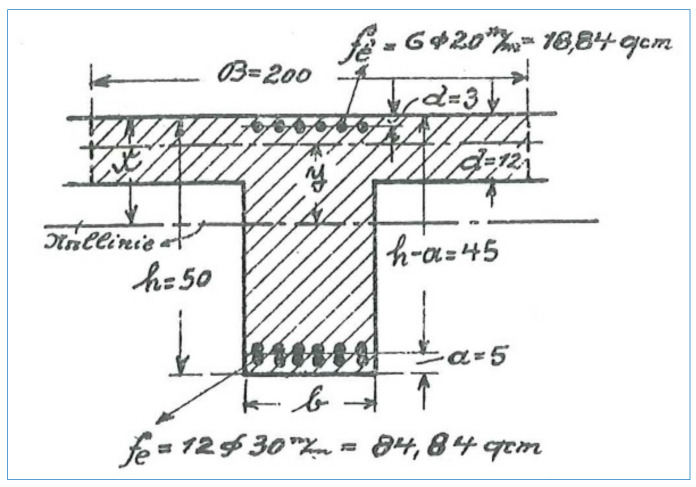
A view of historical structures reinforcement. [33].

The problem was the not fully inventoried landing beam B-2, loaded with two large, concentrated forces originating from the reaction of the B-1cheek beams. With the existing cross-section, the bottom reinforcement in the form of 8 bars ø18 (equivalent to steel of A-I class) would be required, while only 2 such bars were inventoried. In the period when the staircases structures has been made, the concentration of reinforcement was used, which would be unacceptable at the present time (sketch below according to [33]—Figure 12, 12ø30 in cross-section with b = 20 cm).

At the present times, the width of the beam could not be less than 42 cm. It cannot be ruled out that the existing reinforcement of the B-2 beam has not been fully inventoried or that there is an additional iron (steel) profile inside it. The cross-section of two ø18 iron inserts is F_a_ = 5.05 cm^2^ transfers the moment of ~47.5 kNm with a possible maximum of 177.15 kNm (the required reinforcement is 8ø18 with F_a_ = 20.36 cm^2^). Such a large bending moment could be transferred by the parallelepiped I 200 × 200 (h = 200 mm and b = 200 mm), produced since 1902, which could easily fit into the cross-section of the B-2 beam.

### 3.3. K2 Reinforced Concrete Staircase

**Figure 13:**
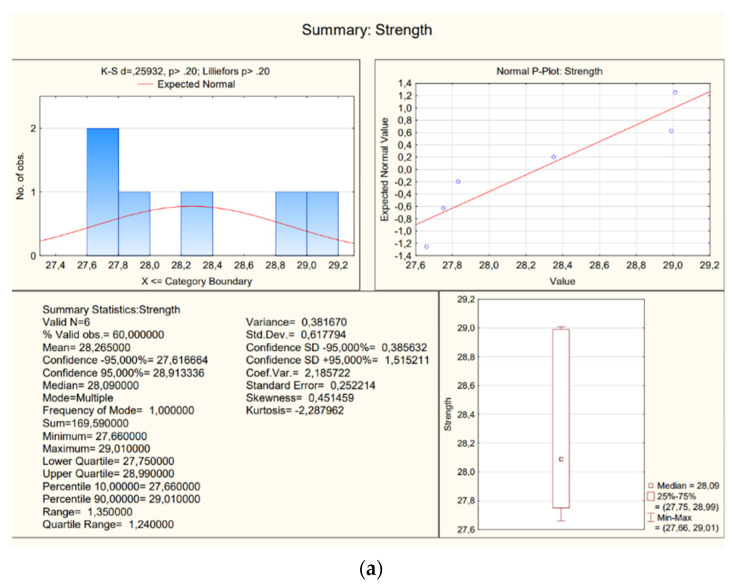

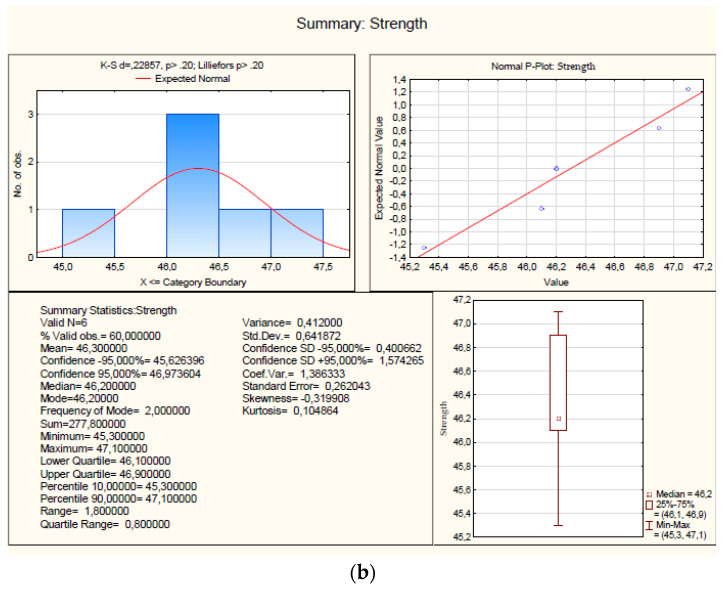
A view of statistics (**a**) samples 8a–f; (**b**) samples 9a–f.

The obtained results are presented in Table 3 and Figure 13—there are two representative samples (samples 1 were taken from the running plate, samples 2 were taken from the landing plate), and each has results a–f. Tests were done on different sub-samples from the same concrete, which were taken in multiple places.

**Table 3:** Strength test results for sample no. 8, 9.

Sample No.	Sample Weight [kg]	Average Height of the Prepared Sample [mm]	Average Length of the Prepared Sample [mm]	Strength on Compression f [N/mm^2^]
8- average	1.45	98	80	28.27
8a	1.46	99	78	28.35
8b	1.44	96	79	29.01
8c	1.47	95	82	27.66
8d	1.42	97	80	28.99
8e	1.46	99	87	27.83
8f	1.45	98	76	27.75
9- average	1.19	98	70	46.3
9a	1.2	98	70	47.1
9b	1.23	96	71	45.3
9c	1.15	99	73	46.2
9d	1.34	97	72	46.9
9e	1.13	98	69	46.1
9f	1.17	99	67	46.2

The P-3 plate rests on both sides of the B-3 running beams’ cheeks and it cannot be ruled out that it is fixed in them. For this slab, the reinforcement was inventoried in the form of 7 bars ø8/per 1 m width (equivalent of A-I class steel) with F_a_ = 3.59 cm^2^. This spacing (every 14 cm) is sufficient to carry the expected loads, therefore no additional reinforcements are provided for here. The situation is similar with the construction of the B-3 beam. It was recommended that, prior to the commencement of construction works, the size and spacing of the reinforcement inserts should be confirmed in an amount not less than 4ø16 (equivalent to A-I class steel) with F_a_ = 7.40 cm^2^.

## 4. A Proposal to Strengthen the Existing Structures

A very interesting element of the research was the assessment of over 100-year-old structures, especially the reinforced concrete. It was a pioneering period in the use of reinforced concrete. Despite the fact that the requirements then for the strength classes of materials, diameters and spacing of reinforcement inserts were significantly different from the present ones, the over 100-year service life of these structures proves that the solutions were properly adopted at the beginning of the 20th century. The authors of the research, wanting to keep the existing structures unchanged (to maintain their authenticity), without additional visible interference, proposed to strengthen them by implementing FRCM (Fibre Reinforced Cementitious Matrix) composite systems using carbon mesh of ceramic structures) and PBO (polyparaphenylene benzobisoxazol) mesh (for reinforced concrete structures). Both types of the nets differed from each other by the terms of strength, density and spacing of the carrying fibers. Carbon meshes were characterized by the same spacing of fibers and the same load capacity in two orthogonal directions (it is important for tensile stresses in two directions in the stair vault—diagrams of the distribution of internal forces and stresses in the vault Figure 10). For PBO meshes, the main direction of the load-bearing capacity was determined and the associated much higher fiber density was associated with it. Both carbon mesh and PBO mesh should be attached to properly prepared substrates in inorganic matrices and, if necessary, anchored with system connectors.

The FRCM technology using carbon mesh and PBO mesh is now more and more commonly used to strengthen masonry and reinforced concrete structures, especially those historic ones. A suitable example of this is central Italy, located in the seismic impact zone, where the strengthening of the structure with the “FRCM technology” allowed the preservation of valuable immovable monuments even after several earthquakes. Such solutions, apart from their strengthening function, should be at the same time not noticeable enough not to disturb the aesthetics of the visual reception of the rooms in which they are used, and thus their functional comfort. The thickness of a single reinforcement layer does not exceed 1 cm, and at the same time it can be an additional fire protection for shallowly laid reinforcement, because without losing its load-bearing parameters, such protections can withstand temperatures of 550 °C to 600 °C. This regards the application of meshes only on mineral matrices, but not to joints using epoxy resins. Additional finishing layers, such as plasters, can be applied on the reinforcement. Before the application of FRCM reinforcements, the pull-off strength research of the substrate should be checked (this applies to the adhesion of future matrices). It should be not less than 1.5 MPa. The test should be carried out using the so-called Tear-off mushrooms by gluing them with epoxy glue to the smooth and cleaned surface, circumferential incision of the substrate and then tearing them off using a tear-off device, e.g., Positech AT-M. After starting the works, the wall load-bearing capacity should be assessed in the places of concentrated loads, i.e., in the places where the stringers, landings and platforms are supported. Such checking and possible strengthening of wall fragments would also occur when new stair structures rest on them. The application of the meshes should be preceded by preparatory works with the use of an appropriate concrete or construction ceramics re-profiling system when the substrate is uneven, peeling and with cavities. The system described above is used to strengthen reinforced concrete elements, mainly in the areas of bending, shear and torsional stresses. Strengthening the stair elements should be performed on the entire surfaces qualified for this purpose with the above-mentioned PBO composite meshes. As is the case with the reinforcement of masonry structures with carbon meshes, the application of PBO meshes must be preceded by consultation with a technical specialist of the system application, who will indicate to the contractor all the detailed requirements for its use in the case in question and confirm in writing that the application of the structure reinforcement system has been completed in accordance with all the requirements set by the manufacturer in the technical data sheets. It should be performed under strict supervision of authorized persons (works manager, supervision inspector).

## 5. Discussion and Conclusions

**Figure 14:**
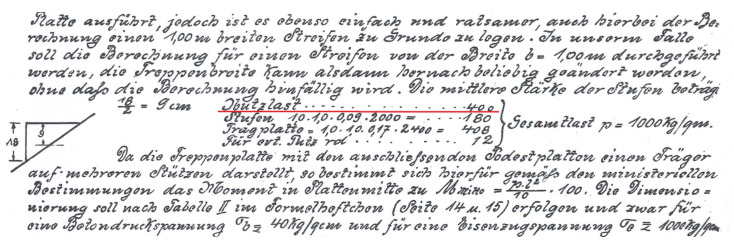
The fragment of calculations from publication [33]. Translation below.

A comprehensive diagnostic analysis of a building structure often requires the participation of people with appropriate qualifications and experience, which will allow for appropriate orientation and overall assessment of the problem under consideration [1,2,3,4,5,6,7,8,9,10,11,12,13,14,18,19,35,36,37,38,39,40,41,42,43,44,45,46,47,48,49,50,51,52,53,54]. The construction materials discussed in the article are undoubtedly of valuable historical value, especially as they come from the pioneering period of implementing concrete and iron (steel) connections on the European construction market. Their valuable element is also a different computational and constructional approach to the selection of reinforcement inserts of reinforced concrete elements and their distribution in the concrete cross-section of the elements. Moreover, at the turn of the 19th and 20th centuries, as well as at the beginning of the latter, materials with much lower strength parameters were used, which, in the aspect of current research, may equal the materials currently used, but are not identical to them. A particularly valuable object here are the original ceramic vaulted stairs, for which some concerns may be caused by their load-bearing capacity due to the expected quite high operational load, i.e., 4.0 kN/m^2^, but at the turn of the above-mentioned centuries, the dimensioning of the stair structure was taken into account already functional loads of 4.0 kN/m^2^ (below is a fragment of calculations from 1910 according to [33]—Figure 14- Nutzlast—400 kg/m^2^—including the translation from the German language).

*“If the calculations are to be carried out for a strip with a width of b = 1.00 m, then the width of the stairs can be freely changed without reducing the correctness of the calculations. The average thickness of the steps 18/2 = 9 cm*

*operational load—400 kg/m^2^*

*steps—180 kg/m^2^*

*landing plate—408 kg/m^2^ total load—p = 1000 kg/m^2^*

*plaster—12 kg/m^2^*

*Since the running plates of the stairs together with the landing plates are a load-bearing structure with many sub-pores, according to the guidelines of the Ministry, the bending moment in the middle of the board should be Mśr = pl^2^/10 × 100. Dimensioning should be carried out in accordance with table II in the formula book (page 14 no. 15), specifically for the compressive stress of concrete σ_b_ ≤ 40 kg/cm^2^ and for the tensile stress of iron σ_b_ ≤ 1000 kg/cm^2^.”*

The position of the authors of the publication is to preserve the historical substance to the highest degree of its authenticity, not mentioning about keeping them in general. It is difficult to carry out reliable research and analyzes in terms of determining the safety level of the structures left behind, the computational approach of which was completely different than the current one. Nevertheless, the inspection of the components of the staircases revealed that, despite over 100 years of exploitation, their deformations or scratches are not visually noticeable. So far, no measurements of this type have been carried out. The performed tests and verification calculations allow for the approval of these structures for further operation after taking into account the assumptions and requirements described above.

Structure materials in historic buildings should not be classified as useless in relation to current regulations and standards, only because of their age. In the considered cases, parallel to the laboratory research of concrete and bricks, non-destructive tests were carried out for them using a “Schmidt hammer” sclerometer, which was calibrated on the basis of samples taken and destroyed in the laboratory. The main purpose of these tests was to check the homogeneity of the material structure on the entire surfaces of these structures, which was also confirmed. The quality of the constructions made in year 1840 and in 1910 should be considered as high, and their technical condition as very good.

Currently, science is trying to look ahead towards the development and research of new materials, technologies, conducting advanced laboratory research, but there are still historical objects that are the national heritage of each region, which should be preserved for future generations in full technical efficiency and the highest degree of authenticity. Universal methods of strengthening, repairs and reinforcement have not yet been developed for them. This article not only attempts to justify such a necessity and to justify the preservation of these objects in a version corresponding to the original, but also to maintain their original function, but in the new conditions of the requirements set by building regulations and standards. What would science be if it did not transfer the results of its research to reality, even the one concerning “obsolete” constructions, one of which is over 180 years old and the other two over 110 years old? These are not standard and typical solutions, because such solutions in historic structures are still rare. We are dealing here with three different, and significantly different, technologies of performance of stairs with currently not used parameters, which have been subjected to multi-format researches and analyzes of materials, indicating simple but innovative reinforcement solutions, full responsibility for their implementation and operational safety. Reinforcing composite materials are already a well-known solution, but it mainly concerns the technology of their fixing with resins. As a way to strengthen the weakened parts of the structure, solutions based on the FRCM technology using carbon meshes and PBO have been proposed, which are described in detail in point 4 of the article, which are still in the research phase, especially in terms of their strength depending on adhesion to substrates and have not yet been fixed in standards.

This approach to the problem of historical buildings is a significant part of the sustainable development strategy, because such buildings can still perform their original functions or can be used for other purposes. In this way, the need to deposit post-demolition waste in landfills that are already disappearing or is eliminated. Such solutions do not require the energy that is needed to produce new materials, transportation and erection of new facilities.

## Data Availability

The article is original and previously unpublished.
